# Un syndrome confusionnel révélant un syndrome de Fahr avec hyperparathyroïdie

**DOI:** 10.11604/pamj.2013.14.123.1898

**Published:** 2013-03-29

**Authors:** Souad Rharrabti, Ilhame Darouich, Mohamed Benbrahim, Fawzi Belahsen, Ismail Rammouz, Rachid Alouane

**Affiliations:** 1 Service de psychiatrie centre hospitalier universitaire Hassan II de Fès; 2 Service de neurologie centre hospitalier universitaire Hassan II de Fès, Maroc

**Keywords:** Syndrome confusionnel, syndrome de Fahr, retard mental, épilepsie, hyperparathyroïdie, confusional syndrome, Fahr syndrome, mental retardation, epilepsy, hyperparathyroidism

## Abstract

Le syndrome de Fahr est une entité anatomo-clinique rare, caractérisée par des calcifications intracérébrales bilatérales et symétriques, localisées dans les noyaux gris centraux, le plus souvent associées à des troubles du métabolisme phosphocalcique. L'hypoparathyroïdie, primitive ou postopératoire, est l'anomalie la plus classique. L'hyperparathyroïdie est exceptionnellement rapportée comme cause du syndrome de Fahr. Nous rapportons le cas d'une fille de 17 ans suivie depuis l’âge de 12 ans pour une épilepsie avec la notion d'un retard mental depuis l'enfance, qui a présenté un syndrome confusionnel révélant un syndrome de Fahr avec la particularité de l'existence d'une hyperparathyroïdie.

## Introduction

Le syndrome de Fahr est une entité anatomo-clinique rare, caractérisée par des calcifications intracérébrales bilatérales et symétriques, localisées dans les noyaux gris centraux, le plus souvent associées à des troubles du métabolisme phosphocalcique. Nous rapportons le cas d'une fille de 17 ans qui a présenté un syndrome confusionnel révélant un syndrome de Fahr.

## Patient et observation

**Figure 1:**
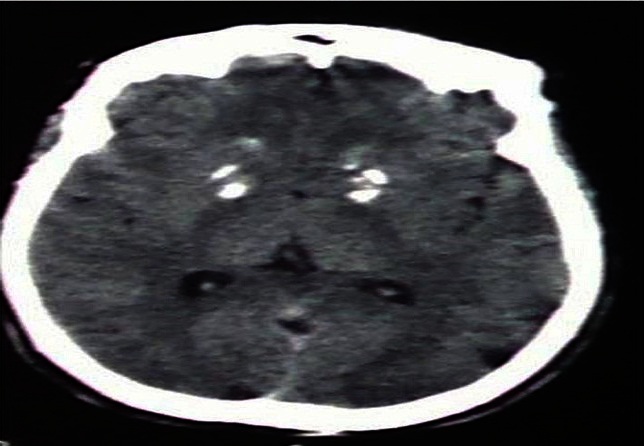
Hyperdensité spontanée bipallidale

Mlle M.S, âgée de 17ans, issue d'une grossesse de déroulement normal. L'accouchement a eu lieu à domicile, sans notion de souffrance néonatale. L'acquisition de la marche a eu lieu à un an, la parole à deux ans et le contrôle sphinctérien à 2 ans. Un retard mental a été constaté par la famille à l’âge de 6ans, et qui ne lui avait pas permis la poursuite de sa scolarité. Depuis l’âge de 12 ans, la patiente a présenté des crises tonico-cloniques généralisées précédées par des hallucinations visuelles. Elle a été mise sous Valproate de sodium 200 mg par jour, avec une bonne évolution clinique. Depuis 1 an, les crises sont devenues de plus en plus fréquentes, d'où l'association de Valproate de sodium avec du phénobarbital. A noter qu'il n'y a pas de cas similaires dans la famille. La patiente a été admise aux urgences psychiatriques dans un état d'agitation psychomotrice. La famille a rapporté la notion de crises convulsives subintrantes avec trouble de la conscience quelques jours auparavant, puis la patiente est devenue instable et insomniaque. L'examen psychiatrique a trouvé une patiente instable, déambulant dans la salle d'examen, désorientée dans le temps et l'espace, avec un regard figé, perplexe, ne répond pas aux questions et apyrétique, l'examen neurologique n'a pas objectivé de raideur méningée, ni de déficit sensitivomoteur. Les réflexes ostéotendineux étaient présents et symétriques, un tonus normal, sans trouble de la coordination, ni d'atteinte des nerfs crâniens. Un scanner cérébral sans et avec injection du produit de contraste a montré une hyperdensité spontanée bipallidale (Figure 1). L’électroencéphalogramme était normal. Le bilan biologique a montré une Hémoglobine à 15,10g/dl, Globules blancs: 8600/mm^3^, Plaquettes: 421000/mm^3^, VS: 4mm/10mm, glycémie: 0,98g/l, urée: 0,22g/l, créatinine: 9mg/l, Na+: 146 mEq/l, K+: 3,6mEq/l. Une PL a montré: ^ème^ et la 6^ème^ décennie de la vie, mais des cas de début plus précoce ont été décrits [2]. Sur le plan histologique, le SF correspond à des dépôts minéraux de la paroi des vaisseaux des noyaux gris centraux (NGC), dont le mécanisme reste mal élucidé [1, 3]. Les calcifications peuvent être physiologiques chez l'enfant dans certaines localisations: faux du cerveau, glande pinéale et plexus choroïdes. Au niveau des NGC, elles sont toujours pathologiques et représentent 15% des calcifications intracérébrales de l'enfant [4]. Les manifestations cliniques du syndrome de Fahr comportent en premier lieu des signes neuropsychiatriques: troubles psychiatriques: troubles du comportement, syndrome confusionnel ou délirant. D'autres manifestations neurologiques sont possibles mais moins habituelles, comme des troubles cognitifs, une détérioration intellectuelle, un retard mental, une atteinte extrapyramidale, des crises convulsives généralisées ou partielles, plus rarement un syndrome pyramidal et une hypertension intracrânienne [5]; lésions dermatologiques liées au déficit en parathormone [6].

Le syndrome de Fahr est le plus souvent associé à une dysparathyroïdie: l'hypoparathyroïdie, primitive ou postopératoire, est l'anomalie la plus classique associant hypocalcémie, hyperphosphorémie, hypocalciurie, hypophosphaturie et diminution du taux sérique de la parathormone.

La pseudohypoparathyroïdie est une affection familiale définie par une résistance périphérique à la parathormone. Sur le plan biologique, on retrouve alors une hypocalcémie, une hyperphophorémie, mais un taux sérique de la parathormone normal ou élevé. L'hyperparathyroïdie est exceptionnellement rapportée comme cause du syndrome de Fahr [7–9]. La particularité de notre observation est la survenue du syndrome de Fahr à un âge précoce, le mode de début était sous forme d'un retard mental avec des crises convulsives généralisées, le syndrome confusionnel était révélateur et l'association à une hyperparathyroïdie. L'analyse de la littérature a permis de colliger moins d'une dizaine d'observations d'hyperparathyroïdie et/ou de pseudohypoparathyroïdie au cours du syndrome de Fahr [5, 8]. D'autres causes donnent des calcifications intracérébrales telles que les endocrinopathies, les maladies de système, la maladie c'liaque, les infections, les tumeurs cérébrales primitives ou secondaires calcifiées et d'autres maladies diverses, cependant, au cours de ces différentes pathologies, les calcifications intracérébrales ont des sièges et des aspects différents [10].

Le pronostic du syndrome de Fahr est bon, car les signes cliniques, neuropsychiques régressent après correction des perturbations phosphocalciques [7, 9].

## Conclusion

Le syndrome de Fahr est une entité rare. Le syndrome confusionnel peut être révélateur comme dans le cas de notre patiente. Ce travail souligne l'intérêt de la recherche des troubles du métabolisme phosphocalcique en présence de manifestations neuropsychiatriques associée à des calcifications des noyaux gris centraux, afin de dépister un syndrome de Fahr et d'adopter ainsi, les mesures thérapeutiques les plus appropriées.
